# Supplementary material for: Platelet pannexin-1 channels modulate neutrophil activation and migration but not the progression of abdominal aortic aneurysm
Source: Front Mol Biosci. 2023 Mar 6;10:1111108. doi: 10.3389/fmolb.2023.1111108 (PMC10025481; doi:10.3389/fmolb.2023.1111108)
Supplement: Supplementary file 2 [file DataSheet1.PDF]

Supplementary Material

1 Supplementary Figures and Tables

1.1 Supplementary Figures

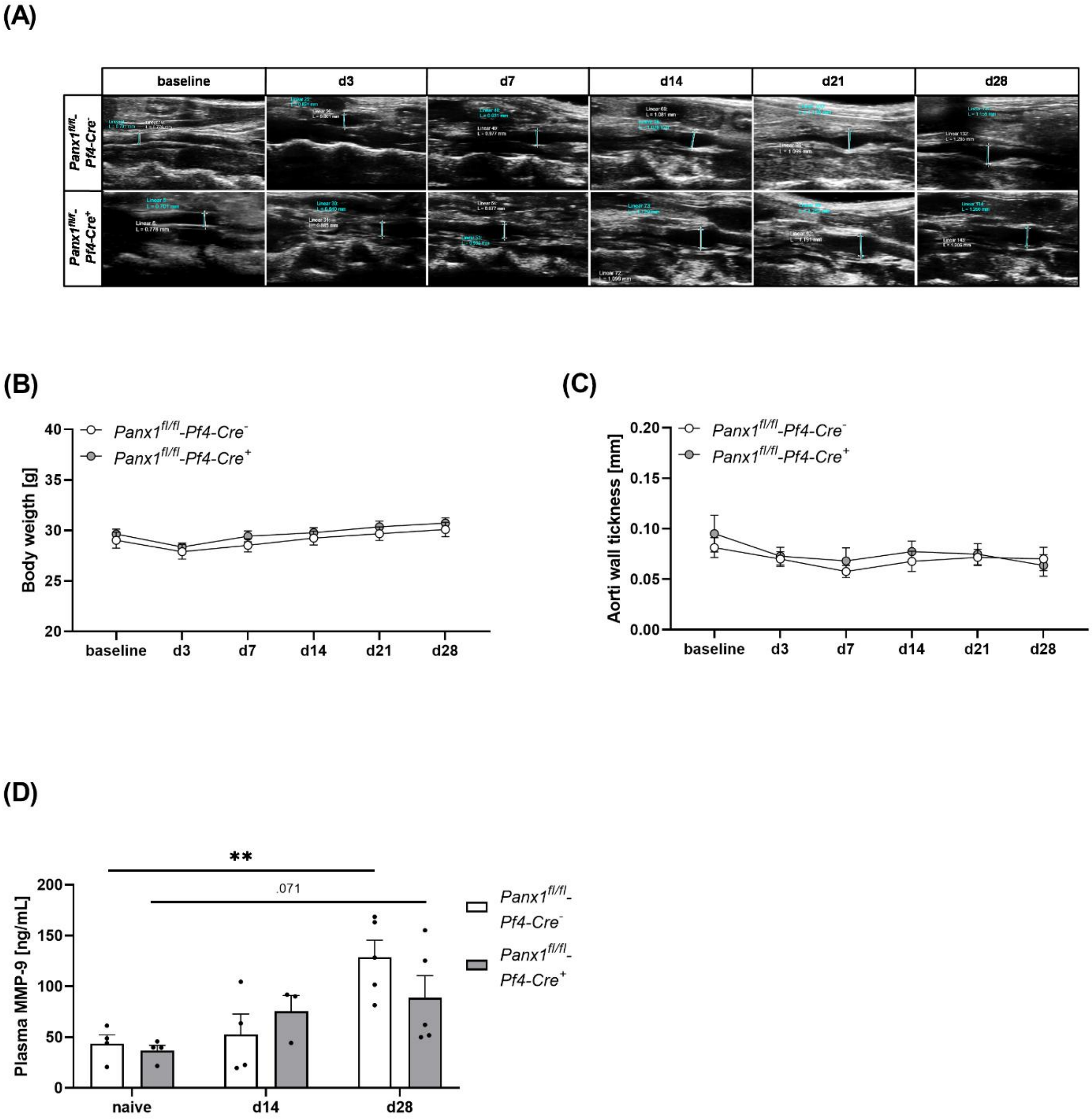

Supplementary Figure 1. Platelet specific Panx1 deletion leads to unaltered aortic wall thickness during AAA development after PPE surgery. **(A)** Representative Ultrasound images of the abdominal aorta from Panx1 WT (Panx1 fl/fl PF4-Cre$^-$; n=11) and Panx1 KO (Panx1 fl/fl PF4-Cre$^+$; n=7) mice at day 28 after PPE perfusion. Ultrasound imaging was conducted day 0 (pre surgery) and at day 3, 7, 14,

21 and 28 after PPE. **(B)** Body weight of Panx1 WT (n=11) and Panx1 KO (n=7) mice during PPE induced AAA formation. **(C)** Aortic wall thickness progression within the aneurysm segment in Panx1 WT (n=11) and Panx1 KO (n=7) mice over a time period of 28 days after PPE. The aortic wall thickness was determined via ultrasound measurements at the indicated time points; **(D)** MMP9 plasma concentration of PPE operated Panx1 KO mice (n=3-5) at day 14 and day 28, compared to Panx1 WT mice (n=3-5). Plasma of naïve Panx1 WT (n=4) and Panx1 KO (n=4) mice served as control. Data are represented as mean values ± SEM. Statistical analysis was performed using two-way ANOVA with a Sidak's multiple comparisons post-hoc test. AAA = Abdominal aortic aneurysm; MMP-9 = Matrix metalloproteinase-9; Panx1 = Pannexin-1; PPE = Porcine pancreatic elastase perfusion.

.

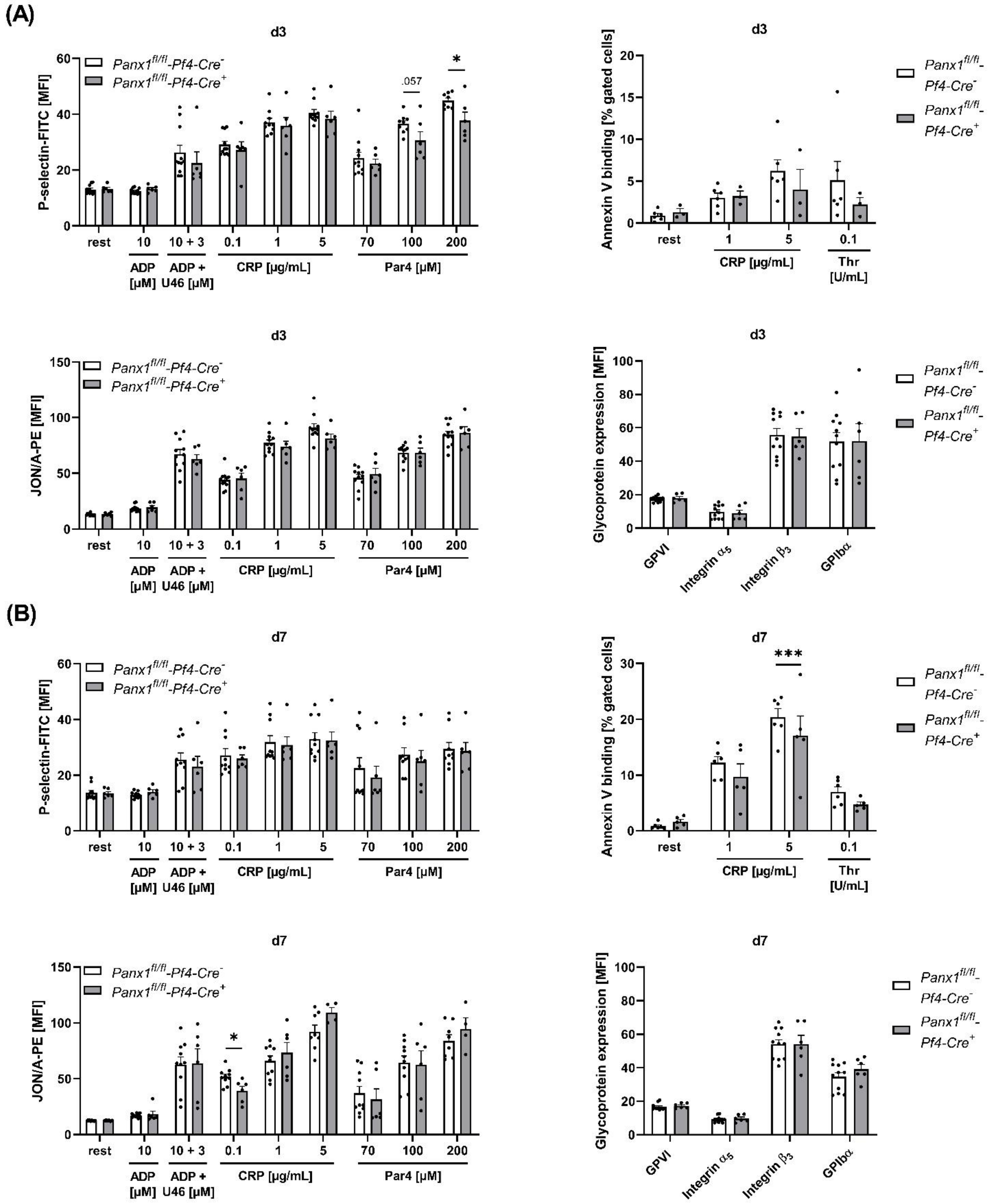

Supplementary Figure 2. Platelets of Panx1 deficient mice reveal an unaltered platelet degranulation, activation and PS-exposure during the inflammatory phase of AAA development. Washed platelets from PPE operated Panx1 WT (*Panx1 fl/fl PF4-Cre*$^{-}$) and Panx1 KO (*Panx1 fl/fl PF4-Cre*$^{+}$) mice were

analysed via flow cytometry at day 3 **(A)**, and at day 7 **(B)** after surgery. **(A-B)** Platelet degranulation (P-selectin-FITC), integrin $\alpha_{IIb}\beta_3$ activation (JON/A-PE) and PS-exposure (Annexin V-Cy5 binding) were analysed after platelet stimulation with indicate agonists. In addition, platelet surface expression of GPVI, integrin α_5, integrin β_3 and GPIbα was analysed via flow cytometry (Panx1 WT n=5-11; Panx1 KO n=4-6). Data are represented as mean values ± SEM. Statistical analysis was performed using an **(A-B)** unpaired multiple student's t-test; $^*p < 0.05$, $^{***}p < 0.001$. ADP = Adenosine diphosphate; CRP = Collagen-related peptide; MFI = Mean fluorescence intensity; Panx1 = Pannexin-1; PAR4 = Protease-activated receptor 4 peptide; PPE = Porcine pancreatic elastase perfusion; Rest = resting; Thr = Thrombin; U46619 (U46) = Thromboxane A_2 analogue.

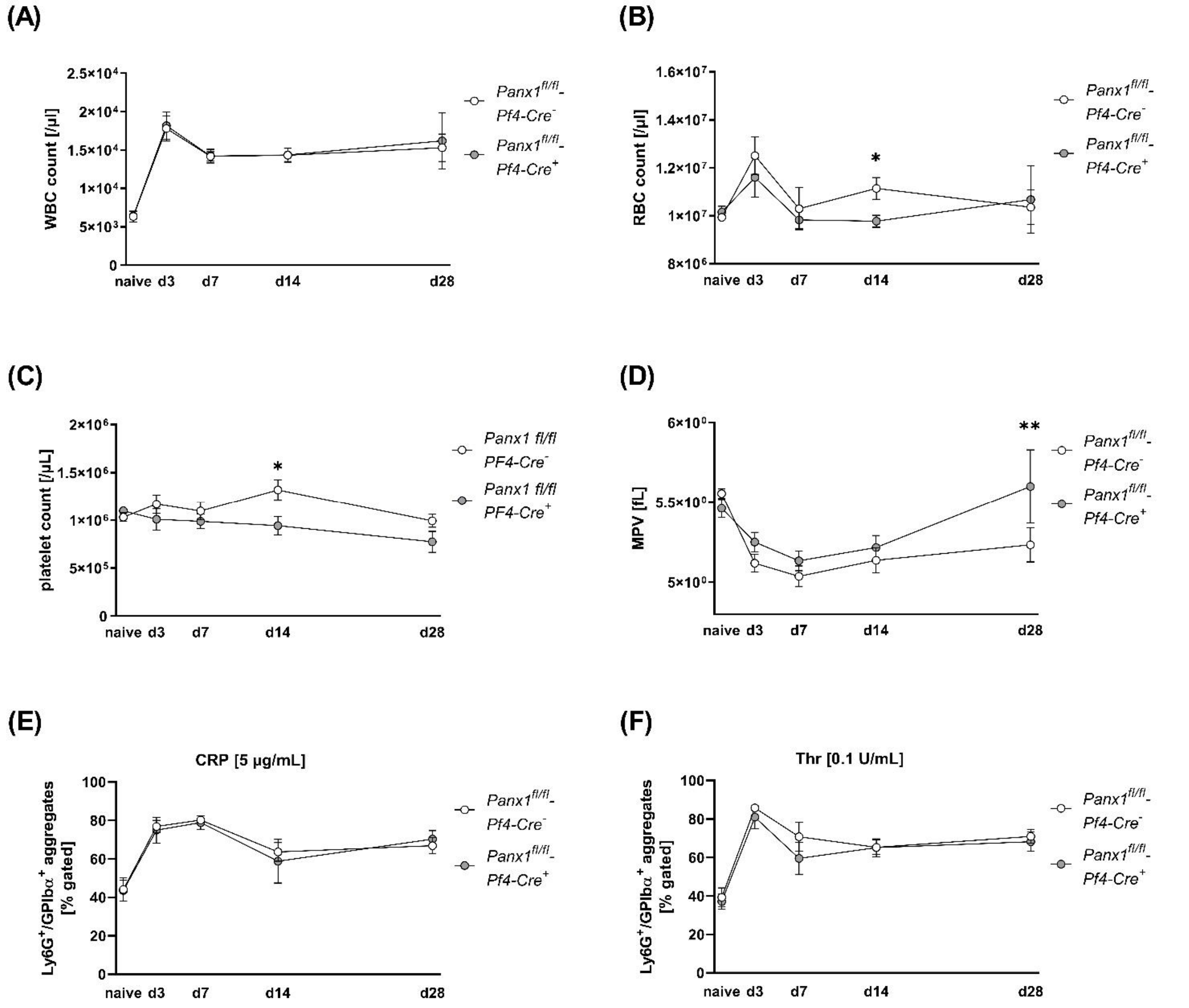

Supplementary Figure 3. Platelet Panx1 deficient mice reveal alteration in blood cell count and MVP during PPE induced AAA formation**. (A)** WBC, **(B)** RBC, **(C)** platelet counts and **(D)** the MPV of Panx1 WT (*Panx1 fl/fl PF4-Cre*$^-$; n=7-11), respectively Panx1 KO (*Panx1 fl/fl PF4-Cre*$^+$; n=5-7) mice were analysed at day 0 (naïve), 3, 7, 14, and day 28 after PPE surgery. **(E-F)** Platelet neutrophil aggregates in PPE operated Panx1 WT (n=6-11) and Panx1 KO (n=4-6) mice after stimulation with **(E)** CRP [5 µg/mL] or **(F)** thrombin [0.1 U/mL]. Aggregate formation was analysed at day 0 (naive), 3, 7, 14 and 28 after PPE surgery via flow cytometry as double positive events for the platelet marker GPIbα (CD42b) and the neutrophil marker Ly6G. Data are represented as mean values ± SEM. Statistical analysis **(A-F)** was performed using a two-way ANOVA with a Sidak's multiple comparisons post-hoc test; *$p < 0.05$, **$p < 0.01$. CRP = Collagen-related peptide; MPV = Mean platelet volume; Panx1 = Pannexin-1; PPE = Porcine pancreatic elastase perfusion; RBCs = Red Blood Cells, Thr = Thrombin; WBCs = White Blood Cells.

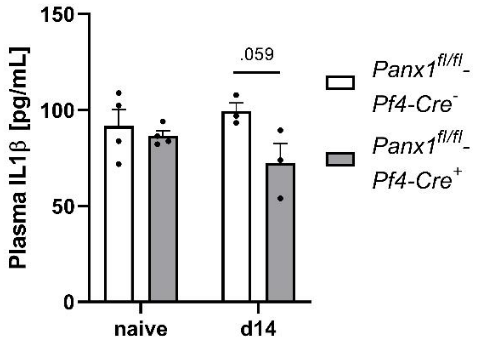

Supplementary Figure 4. Plasma IL1β level are unaltered in platelet Panx1 deficient mice during PPE induced AAA progression. IL1β plasma concentration of PPE operated Panx1 KO mice (n=3-5) at day 14 post surgery, compared to Panx1 WT mice (n=3-5). Plasma of naïve Panx1 WT (n=4) and Panx1 KO (n=4) mice served as control. Data are represented as mean values ± SEM. Statistical analysis was performed using a two-way ANOVA with a Sidak's multiple comparisons post-hoc test; $^*p < 0.05$, $^{**}p < 0.01$, $^{***}p < 0.001$. IL-1β = Interleukin-1β; Panx1 = Pannexin-1; Panx1 = Pannexin-1; PPE = Porcine Pancreatic Elastase.

(A)

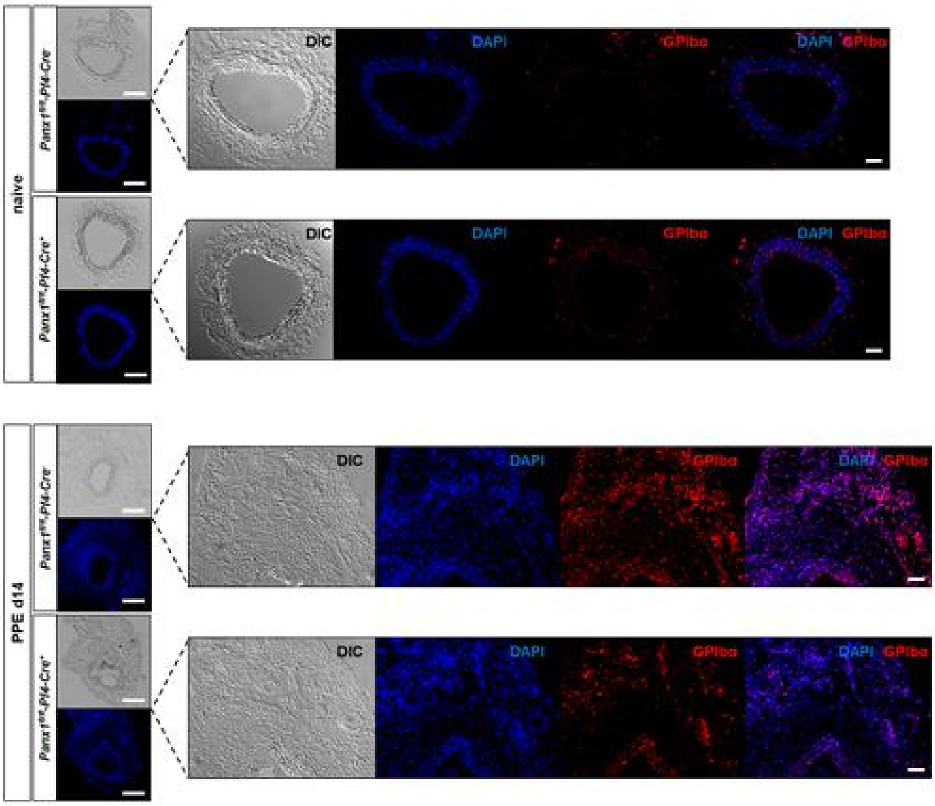
naive
PPE d14
DIC
DAPI
GPIbα
DAPI GPIbα

(B)

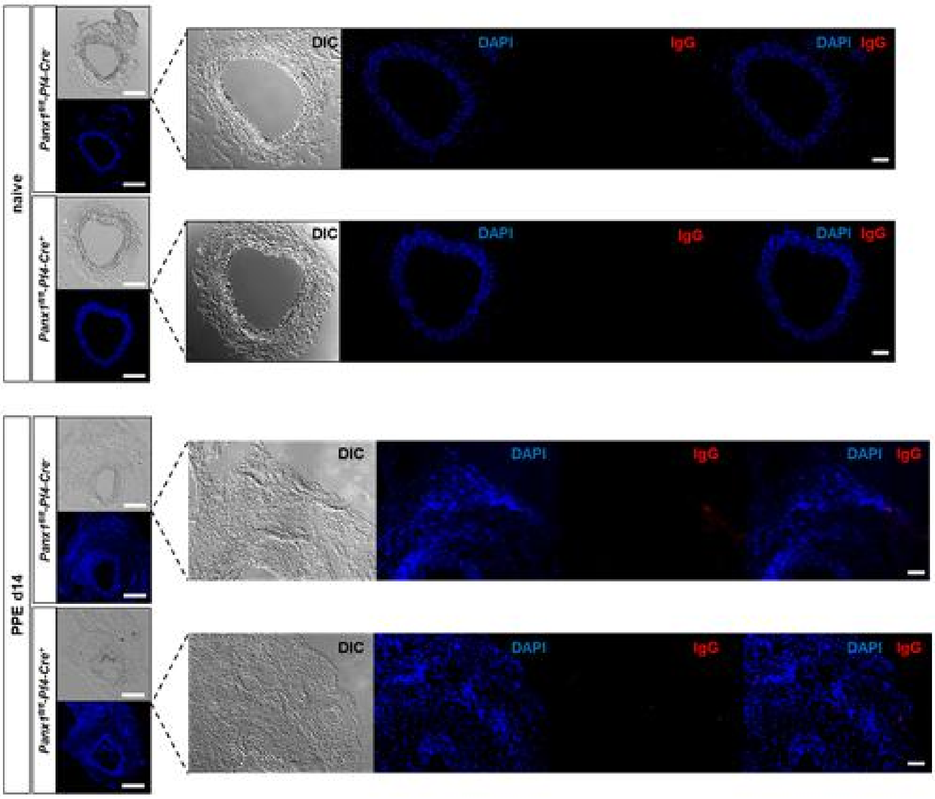
naive
PPE d14
DIC
DAPI
IgG
DAPI IgG

Supplementary Figure 5. Immunofluorescence staining of platelets within the aortic tissue of PPE operated Panx1 deficient mice. **(A)** Immunofluorescence staining of platelets (GPIbα/Cy5; red) in the aortic tissue of Panx1 WT (*Panx1 fl/fl PF4-Cre*$^{-}$; n=3) and Panx1 KO (*Panx1 fl/fl PF4-Cre*$^{+}$; n=3) mice at day 14 after PPE surgery and **(B)** the respective IgG controls (scale bar: 200 µm and 50 µm). Aortic tissue of naïve Panx1 WT (n=3) and Panx1 KO (n=4) mice served as control. DIC= Differential interference contrast; Panx1 = Pannexin-1

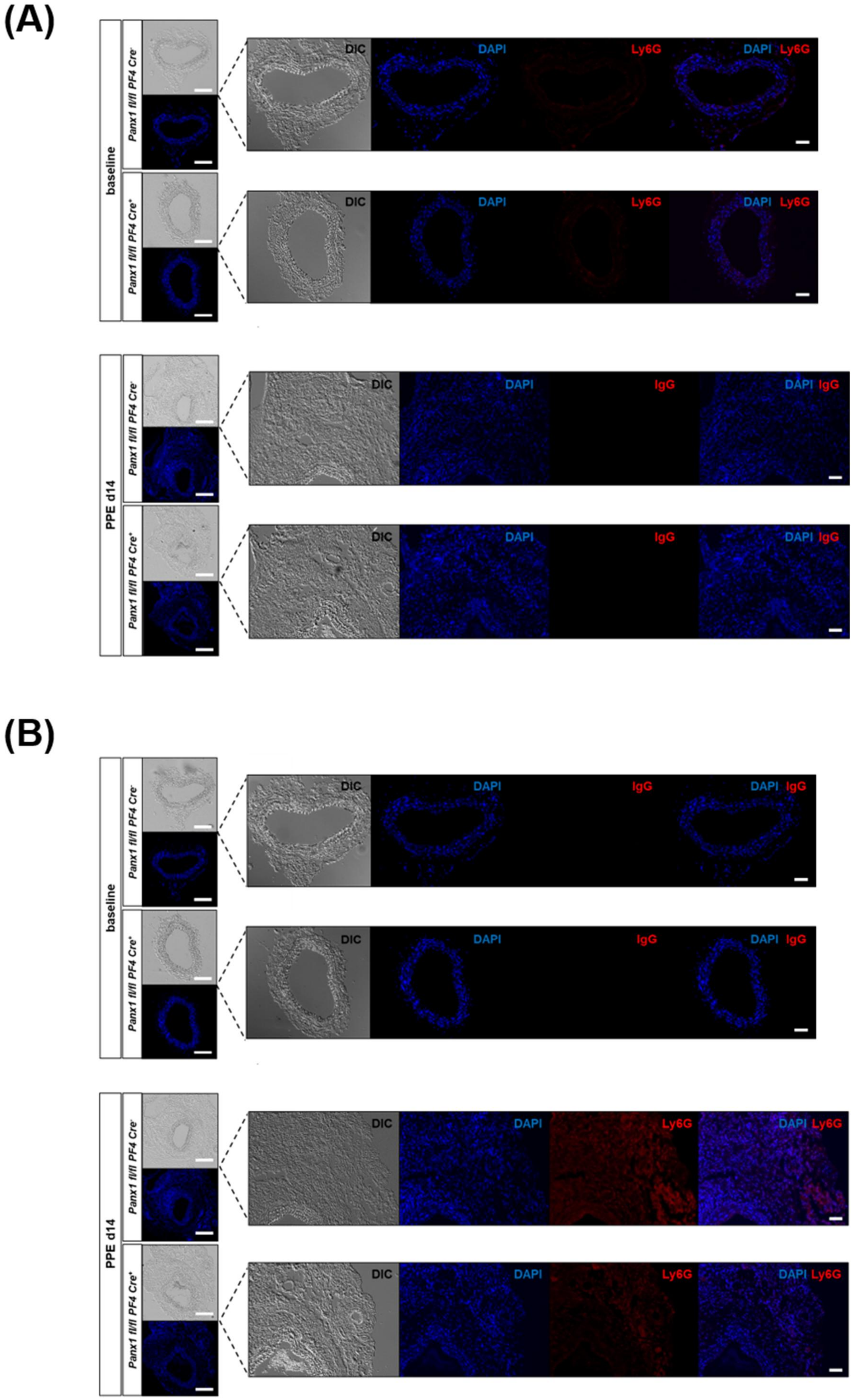
(A)
baseline
Panx1 fl/fl PF4 Cre-
Panx1 fl/fl PF4 Cre+
DIC
DAPI
Ly6G
DAPI Ly6G
PPE d14
IgG
DAPI IgG
(B)

Supplementary Figure 6. Immunofluorescence staining of neutrophils within the aortic tissue of PPE operated Panx1 deficient mice. **(A)** Immunofluorescence staining of neutrophils (Ly6G/Cy5; red) in the aortic tissue of Panx1 WT (*Panx1 fl/fl PF4-Cre*$^-$; n=3) and Panx1 KO (*Panx1 fl/fl PF4-Cre*$^+$; n=3) mice at day 14 after PPE surgery and **(B)** the respective IgG controls (scale bar: 200 µm and 50 µm). Aortic tissue of naïve Panx1 WT (n=3) and Panx1 KO (n=4) mice served as control. DIC= Differential interference contrast; Panx1 = Pannexin-1
